# Spontaneous Coronary Artery Dissection in a Female Patient With Acute Myocardial Infarction

**DOI:** 10.7759/cureus.31839

**Published:** 2022-11-23

**Authors:** Varinder Bansro, Mamoon Al-Afun, Anusha K Battula, Tekabe N Birhane, Rajendra Shetty

**Affiliations:** 1 Internal Medicine, University of Maryland Capital Region Health, Largo, USA; 2 Interventional Cardiology, University of Maryland Capital Region Health, Largo, USA

**Keywords:** antiplatelet therapy, coronary angiography, fibromuscular dysplasia, acute myocardial infarction, spontaneous coronary dissection

## Abstract

Spontaneous coronary artery dissection (SCAD) is the formation of a false lumen within a vessel wall, with an accumulation of blood leading to vessel occlusion, mimicking symptoms of acute myocardial infarction (AMI). Here, we discuss the case of a middle-aged woman with STEMI who was found to have coronary artery dissection on coronary angiography during left heart catheterization. Clinicians should have a high suspicion of SCAD in young females presenting with AMI without traditional risk factors for coronary artery disease. Such patients should receive urgent angiography. Once the diagnosis is confirmed, there are no clear guidelines for treating AMI secondary to SCAD. Hemodynamically stable patients can be managed with the immediate initiation of antiplatelet therapy and beta-blockers. Thrombolytic therapy is avoided due to the risk of dissection and intramural hematoma. Coronary artery bypass graft (CABG) is indicated in patients with multiple vessel involvement or patients who have had a primary coronary intervention fail. Bioresorbable vascular scaffolds (BVS) may be a better option in STEMI or hemodynamic instability. However, current treatment strategies are based on expert opinion and a few case studies.

## Introduction

Acute coronary syndrome (ACS) is caused by a mismatch between oxygen consumption and demand by the myocardium. It usually causes secondary plaque rupture, which leads to vessel occlusion. Spontaneous coronary dissection is defined as a non-atherosclerotic, non-traumatic, non-iatrogenic cause of acute coronary artery syndrome. It forms a false lumen within the arterial wall, resulting in intramural hematoma and gradual lumen stenosis, causing symptoms like ACS [1]. The incidence rate of spontaneous coronary artery dissection (SCAD) is 1-4% of all cases of acute coronary syndrome [1-2]. This rate increases to 25-30% in young and middle-aged women coming in with ACS [3]. Predisposing factors that are associated with SCAD are fibromuscular dysplasia (FMD), female gender, pregnancy-related factors, possibly hormonal therapy, mixed connective tissue disorders, and inflammatory disorders [2]. We present the case of a middle-aged woman who came to the ED with STEMI and was found to have coronary artery dissection on coronary angiography.

## Case presentation

A 52-year-old female presented to the emergency department with complaints of mild epigastric pain starting four to five hours before arrival. She described the pain as burning in nature, mild to moderate in intensity, and non-radiating. It was not associated with diaphoresis or shortness of breath. She also endorsed nausea and a few episodes of non-bilious/non-bloody vomiting. She was diagnosed with hypertension a few years ago but has not taken any medication for the last year. The patient admitted to smoking marijuana 3-4 times a week. There was no remarkable family history.​

**Figure 1:**
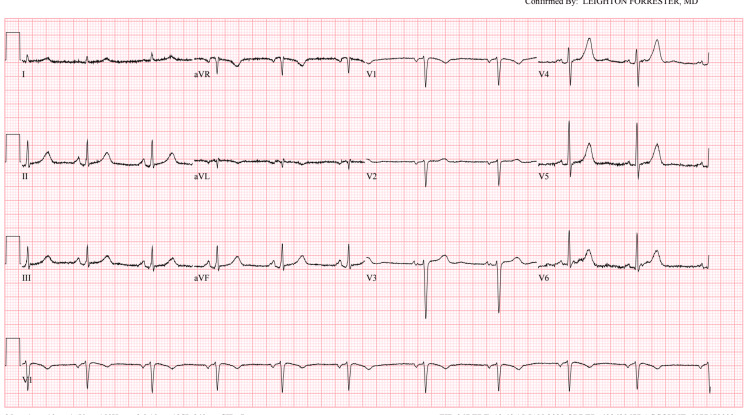
EKG on admission

When she arrived at the ED, her blood pressure was 182/90 mmHg and her heart rate was 72 beats per minute. Physical examination did not reveal any remarkable findings except mild epigastric tenderness. Figure 1 shows the electrocardiogram (EKG) on admission, which shows normal sinus rhythm without any significant ischemic changes. Troponins and chest imaging were normal. The lipid panel shows total cholesterol 311 mg/dl (120-200 mg/dl), triglycerides 108 mg/dl (1-200 mg/dl), HDL 78 mg/dl (>56 mg/dl), and LDL 208 mg/dl (8.5-99 mg/dl). CT abdomen revealed a 10 mm saccular aneurysm arising from the mid-to-distal portion of the right main renal artery. We consulted the vascular surgery team, and they recommended no acute intervention. We admitted the patient for observation.​

**Figure 2:**
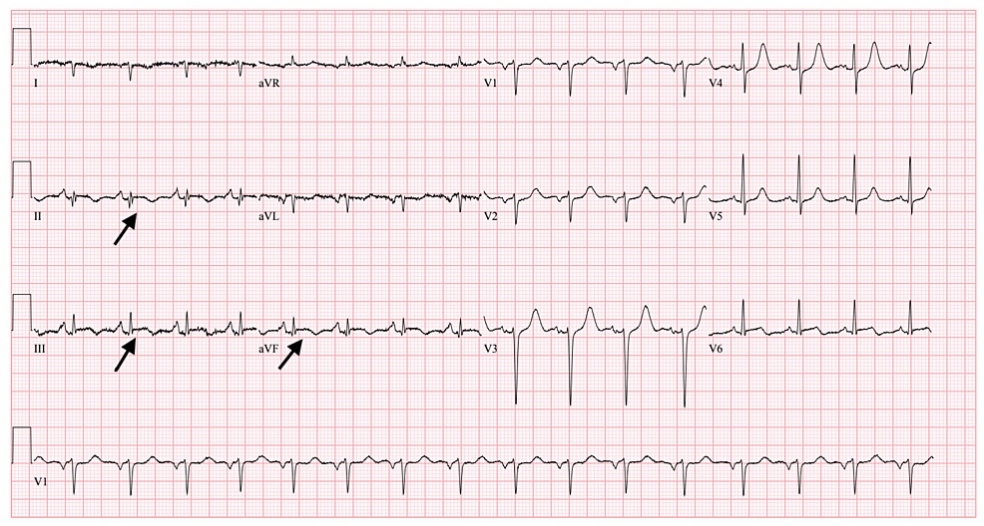
ST elevation in leads II, III and aVF

Subsequently, her next troponin, eight hours later, increased to 1.100 (normal range 0.010-0.029 ng/ml). Figure 2 shows a repeat EKG eight hours later showing subtle ST elevation in inferior leads and left axis deviation. At this point, the patient had similar complaints of epigastric pain and nausea. Code STEMI was activated. A loading dose of aspirin and ticagrelor was given, and the patient was immediately taken to the cath lab.

**Figure 3:**
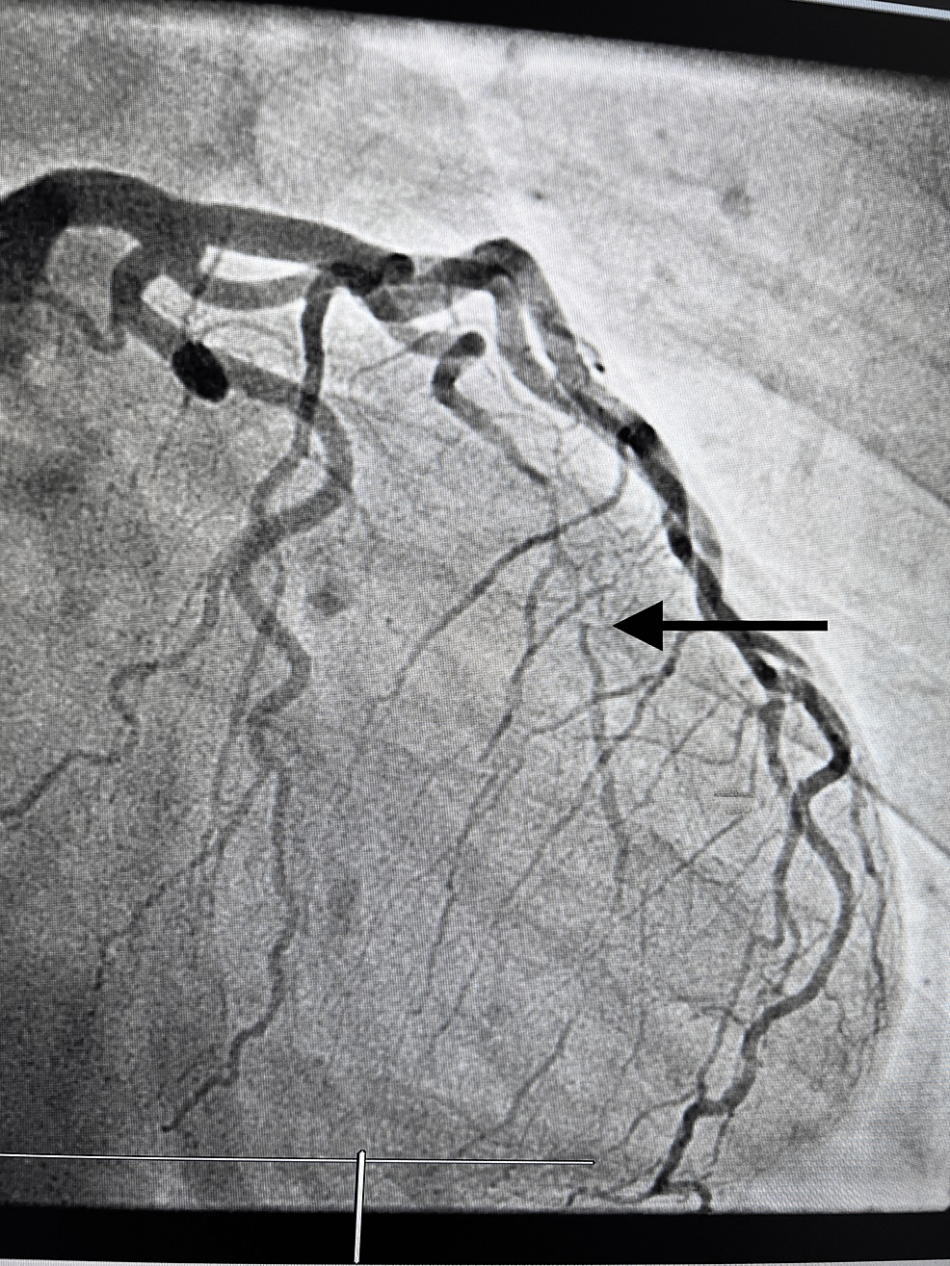
Left heart cath showing type 2 coronary artery dissection in Ramus intermedius artery

A left heart catheterization with selective coronary angiography was performed. As shown in Figure 3, the left heart cath shows the Ramus intermedius appearing to be angiographically normal in the proximal to mid-segment and totally occluded in the mid-segment with trickle flow in the distal segment, which is suspicious for a type 2 spontaneous coronary artery dissection. Echocardiography revealed an adequate left ventricular ejection fraction of 60-65% and normal LV size and thickness. She was started on aspirin and beta-blockers.

We considered the possibility of fibromuscular dysplasia given the renal artery aneurysm on the CT abdomen, hypertension, and spontaneous coronary artery dissection. MRA of the head, chest, abdomen, and pelvis was done. The MRA abdomen revealed an irregular contour with narrowing proximally and a subtle beaded appearance in the mid-segment. 8 mm splenic and 11 mm right renal artery aneurysms were noted. A slight contour irregularity of the left renal artery near the hilum was also present.​ MRA head, neck, and chest did not show any evidence consistent with fibromuscular dysplasia.

Two days after admission, the patient was discharged home on aspirin, carvedilol, atorvastatin, and lisinopril. She was advised to have a close follow-up with the cardiologist. At one and five months of follow-up, she reported no new symptoms.​

## Discussion

SCAD is a condition that occurs after a tear in a blood vessel, leading to the development of a false lumen within the vessel wall, subsequently leading to the accumulation of blood and occlusion of the vessel [4]. This leads to symptoms mimicking myocardial infarction. It is usually non-iatrogenic and not associated with trauma or atherosclerosis [5]. SCAD is emerging as a significant cause of acute coronary syndrome, particularly among young and pregnant women with no or few atherosclerotic risk factors [2,6]. SCAD was a rare entity in the past, but the recent use of higher-sensitivity troponin assays and more access to angiography in ACS has led to increased recognition of this condition (Table 1).

**Table 1:** Types of spontaneous coronary artery dissection Adapted from Saw J. Coronary angiogram classification of spontaneous coronary artery dissection [7].

Type	Description
1	Evident arterial wall stain; Pathognomic angiographic appearance of SCAD.
2	Diffuse stenosis; is often missed. Can vary in severity from mild stenosis to complete occlusion.
3	Mimics atherosclerosis.

Most patients do not have all or only minor cardiovascular risk factors. FMD has a solid association with SCAD. In a study, about 86% of cases of SCAD were associated with FMD of >1 non-coronary artery [8]. In most cases, patients primarily present with ACS and elevated cardiac enzymes. ST elevation is present in 26-87% of cases, whereas 13-69% of patients come in with NSTEMI [6]. Predominant symptoms on presentation are chest pain, but atypical features like nausea, vomiting, dyspnea and back pain are reported in a minority of cases [9].

Urgent angiography should be considered in patients with a high suspicion of SCAD. Diagnosis can be made with angiography in the case of type 1 SCAD. If type 1 is not evident, modalities like OCT (optical coherence tomography) or IVUS (intravascular ultrasound) can be used to confirm the presence of type 2 and type 3 SCAD [10].

Guidelines for the treatment of acute myocardial infarction (AMI) secondary to SCAD are limited. As most cases of SCAD recover to normal coronary architecture within 30 days, most interventionalists stick to conservative management [6]. A recently published retrospective study reported that 84.4% of cases were managed conservatively, suggesting conventional therapies as first-line management for SCAD [11].

Hemodynamically stable patients can be managed medically with antiplatelet therapy (aspirin and clopidogrel) and beta-blockers. Empiric dual antiplatelet therapy benefits by preventing prothrombotic changes and should be started immediately once the diagnosis is confirmed [12]. Beta-blockers help by lowering blood pressure and heart rate and thus reducing shear force on the arterial wall, which prevents the extension of distension [13]. The role of statins is uncertain as there are limited studies of their use in SCAD.

Thrombolytic agents are avoided in the management of SCAD because of the risk of extension of dissection and intramural hematoma [13]. A retrospective study shows that 60% of patients with SCAD who received thrombolytic agents required either rescue percutaneous coronary intervention (PCI) or coronary artery bypass graft (CABG) [14].

PCI is generally the cornerstone for managing ACS, but it is associated with higher rates of complications in SCAD, including the risk of iatrogenic dissection and abrupt vessel occlusion [15]. CABG is reserved for patients who have failed PCI or have high-risk dissection (e.g., left main stem dissection) [16].

Bioresorbable vascular scaffolds (BVS) are a new category of device that can potentially improve the treatment of coronary artery disease [17]. BVS generally act similarly to permanent drug-eluting stents when they are deployed. However, they will disappear within three years after restoring the native vessel state [18]. Therefore, BVS should be considered as a therapeutic option for the treatment of SCAD, where spontaneous healing is part of the natural history of this pathological phenomenon [19].

## Conclusions

SCAD is an often overlooked diagnosis as it mimics an AMI. While it was a rare occurrence in the past, it is being diagnosed more now due to better troponin assays and the availability of angiography. Currently, there are no clear guidelines for treating SCAD. The current treatment recommendations have not been standardized into care and are based on expert opinion and limited data from case studies. The treatment usually initiated is conservative management with antiplatelet therapy and beta-blockers, with the role of statins uncertain. A review of such studies shows the key to treatment is determining the status of coronary artery blood flow. It is imperative to discern between SCAD and CAD as the cause of MI because treatment for CAD can be harmful to SCAD. Thrombolytic agents and PCI, which are standard care for CAD, are associated with higher rates of complications in SCAD. BVS is a new category of device that can potentially improve the outcome of SCAD. However, they need robust studies to determine their efficacy and long-term outcomes.

## References

[1] Saw J, Mancini GB, Humphries KH (2016). Contemporary review on spontaneous coronary artery dissection. J Am Coll Cardiol.

[2] Saw J, Aymong E, Mancini GB, Sedlak T, Starovoytov A, Ricci D (2014). Nonatherosclerotic coronary artery disease in young women. Can J Cardiol.

[3] Nishiguchi T, Tanaka A, Ozaki Y (2016). Prevalence of spontaneous coronary artery dissection in patients with acute coronary syndrome. Eur Heart J Acute Cardiovasc Care.

[4] Alam M, Akah O, Khan T, Zarrar R (2021). Spontaneous coronary artery dissection in patients with fibromuscular dysplasia. Cureus.

[5] Hayes SN, Kim ES, Saw J (2018). Spontaneous coronary artery dissection: current state of the science: a scientific statement from the American Heart Association. Circulation.

[6] Elkayam U, Jalnapurkar S, Barakkat MN, Khatri N, Kealey AJ, Mehra A, Roth A (2014). Pregnancy-associated acute myocardial infarction: a review of contemporary experience in 150 cases between 2006 and 2011. Circulation.

[7] Saw J (2014). Coronary angiogram classification of spontaneous coronary artery dissection. Catheter Cardiovasc Interv.

[8] Saw J, Ricci D, Starovoytov A, Fox R, Buller CE (2013). Spontaneous coronary artery dissection: prevalence of predisposing conditions including fibromuscular dysplasia in a tertiary center cohort. JACC Cardiovasc Interv.

[9] Luong C, Starovoytov A, Heydari M, Sedlak T, Aymong E, Saw J (2017). Clinical presentation of patients with spontaneous coronary artery dissection. Catheter Cardiovasc Interv.

[10] Tweet MS, Gulati R, Williamson EE, Vrtiska TJ, Hayes SN (2016). Multimodality imaging for spontaneous coronary artery dissection in women. JACC Cardiovasc Imaging.

[11] Saw J, Starovoytov A, Humphries K (2019). Canadian spontaneous coronary artery dissection cohort study: in-hospital and 30-day outcomes. Eur Heart J.

[12] Choi JW, Davidson CJ (2002). Spontaneous multivessel coronary artery dissection in a long-distance runner successfully treated with oral antiplatelet therapy. J Invasive Cardiol.

[13] Farooq A, Amjad W, Bajwa AU, Yasin H, Ali R, Pervaiz M (2017). Fibromuscular dysplasia with spontaneous coronary artery disease presenting as acute myocardial infarction. Cureus.

[14] Shamloo BK, Chintala RS, Nasur A, Ghazvini M, Shariat P, Diggs JA, Singh SN (2010). Spontaneous coronary artery dissection: aggressive vs. conservative therapy. J Invasive Cardiol.

[15] Prakash R, Starovoytov A, Heydari M, Mancini GB, Saw J (2016). Catheter-induced iatrogenic coronary artery dissection in patients with spontaneous coronary artery dissection. JACC Cardiovasc Interv.

[16] Hayes SN, Tweet MS, Adlam D, Kim ES, Gulati R, Price JE, Rose CH (2020). Spontaneous coronary artery dissection: JACC state-of-the-art review. J Am Coll Cardiol.

[17] Yang X, Ahmed M, Cutlip DE (2017). When to use bioresorbable vascular scaffolds. US Cardiol Rev.

[18] Serruys PW, Onuma Y, Garcia-Garcia HM (2014). Dynamics of vessel wall changes following the implantation of the absorb everolimus-eluting bioresorbable vascular scaffold: a multi-imaging modality study at 6, 12, 24 and 36 months. EuroIntervention.

[19] Ielasi A, Cortese B, Tarantini G (2016). Sealing spontaneous coronary artery dissection with bioresorbable vascular scaffold implantation: data from the prospective "Registro Absorb Italiano" (RAI Registry). Int J Cardiol.

